# SIRT3 protects bovine mammary epithelial cells from heat stress damage by activating the AMPK signaling pathway

**DOI:** 10.1038/s41420-021-00695-7

**Published:** 2021-10-21

**Authors:** Xiao-Chun Sun, Yue Wang, Han-Fang Zeng, Yu-Meng Xi, Hong Lin, Zhao-Yu Han, Kun-Lin Chen

**Affiliations:** 1 College of Animal Science and Technology, Nanjing Agricultural University, 210095 Nanjing, China; 2 Key Laboratory of Crop and Animal Integrated Farming, Ministry of Agriculture, Jiangsu Academy of Agricultural Sciences, 210014 Nanjing, China

**Keywords:** Cell death and immune response, Gene expression

## Abstract

With global warming, heat stress has become an important challenge for the global dairy industry. Sirtuin 3 (SIRT3), an important mitochondrial NAD+dependent decarboxylase and a major regulator of cellular energy metabolism and antioxidant defense, is integral to maintaining normal mitochondrial function. The aim of this study was to assess the protective effect of SIRT3 on damage to bovine mammary epithelial cells (BMECs) induced by heat stress and to explore its potential mechanism. Our results indicate that SIRT3 is significantly downregulated in heat-stressed mammary tissue and high-temperature-treated BMECs. SIRT3 knockdown significantly increased the expression of HSP70, Bax, and cleaved-caspase 3 and inhibited the production of antioxidases, thus promoting ROS production and cell apoptosis in BMECs. In addition, SIRT3 knockdown can aggravate mitochondrial damage by mediating the expression of genes related to mitochondrial fission and fusion, including dynamin-related protein 1, mitochondrial fission 1 protein, and mitochondrial fusion proteins 1and 2. In addition, SIRT3 knockdown substantially decreased AMPK phosphorylation in BMECs. In contrast, SIRT3 overexpression in high-temperature treatment had the opposite effect to SIRT3 knockdown in BMECs. SIRT3 overexpression reduced mitochondrial damage and weakened the oxidative stress response of BMECs induced by heat stress and promoted the phosphorylation of AMPK. Taken together, our results indicate that SIRT3 can protect BMECs from heat stress damage through the AMPK signaling pathway. Therefore, the reduction of oxidative stress by SIRT3 may be the primary molecular mechanism underlying resistance to heat stress in summer cows.

## Introduction

With global warming, the decrease in milk production due to heat stress has become an urgent problem that needs to be resolved. The normal development of mammary tissue determines the lactating function of dairy cows. In a high-temperature environment, oxidative stress in bovine mammary epithelial cells (BMECs) induces mitochondrial damage in the mammary epithelial cells of dairy cows [1], leading to programmed cell death and affecting the expression of key lactation genes, thus reducing the milk performance of the dairy cows [2]. Mitochondrial dysfunction is usually associated with increased reactive oxygen species (ROS) production. Continuous oxidative stress can induce lipid peroxidation by generating large amounts of ROS, which in turn affect the replication and transcription of mitochondrial DNA, reduce mitochondrial function, further damage mitochondrial DNA [3], and result in mitochondrial damage and cell apoptosis [4, 5].

Mitochondria are double-membrane organelles undergoing constant cycles of division and fusion, a process collectively termed as “mitochondrial dynamics” [6]. The overall morphology of the mitochondria changes dynamically according to the physiological conditions of the cells. Mitochondria are the main site of cell oxidative respiration, and their main function is to produce adenosine triphosphate (ATP) through oxidative phosphorylation to maintain cell growth and development. Mitochondrial dysfunction caused by oxidative damage is characterized by morphological changes and functional loss of mitochondria. The destruction of the mitochondrial membrane potential is the main indicator of mitochondrial dysfunction. Moreover, oxidative stress in mitochondria can induce changes to their permeability and trigger the mitochondrial apoptosis pathway. Apoptosis is a type of programmed cell death that can be triggered by external or internal stimuli. Mitochondria play a part in both pathways but are particularly prominent in the internal pathways. There are several triggers that can activate the intrinsic signaling pathway of apoptosis, and the intracellular signals generated by the stimulation of these inducements can directly act on the corresponding target mitochondria in the cell and activate the apoptotic signaling pathway via the mitochondrial pathway. These stimuli cause changes in the mitochondrial intima, leading to the opening of the mitochondrial permeability transition pore (mPTP), triggering changes in the mitochondrial transmembrane potential, and the release of pro-apoptotic proteins from the membrane space into the cytosol [7]. The activation and regulation of apoptosis via the mitochondrial pathway is mediated by members of the Bcl-2 protein family [8].

Sirtuin 3 (SIRT3), a member of the NAD+-dependent deacetylase family, is mainly distributed in the mitochondria [9] and is particularly sensitive to mitochondrial oxidative damage [10]. SIRT3 reduces ROS production by inducing acetylation of mitochondrial proteins at lysine sites and plays an important role in stabilizing mitochondrial structure and function as well as the stress response [11, 12]. SIRT3 can inhibit the opening of the mPTP to stabilize the mitochondrial membrane [13], promote mitochondrial fusion to maintain the number of mitochondria [14], and promote mitochondrial autophagy to eliminate abnormal mitochondria [11]. SIRT3 can regulate the activation and expression of mitochondrial proteins and decrease the production of ROS, which plays an important role in stabilizing mitochondrial structure and function and stress response [15, 16]. Overexpression of SIRT3 can effectively maintain cellular redox balance and promote mitochondrial aerobic phosphorylation [17–19]. AMPK is an important molecule involved in the regulation of energy metabolism in cells. This can sense a low-energy state and promote ATP synthesis accordingly by increasing catabolism and decreasing anabolism to maintain cellular energy metabolism homeostasis [20]. AMPK and its signal network participate in normal energy metabolism of the body, counteract the stress response, and promote the survival of the body [21]. Studies have shown that the SIRT3 and AMPK pathways are closely related to the regulation of mitochondrial antioxidant processes [22, 23].

In view of the probable relationship between SIRT3 and AMPK, we used a high-temperature-induced BMEC heat stress model to study the effect of SIRT3 on oxidative damage and its function in the progression of cells under heat stress conditions and to clarify its regulatory mechanism.

## Results

### SIRT3 shows low expression in heat-stressed bovine mammary tissue samples

**Fig. 1:**
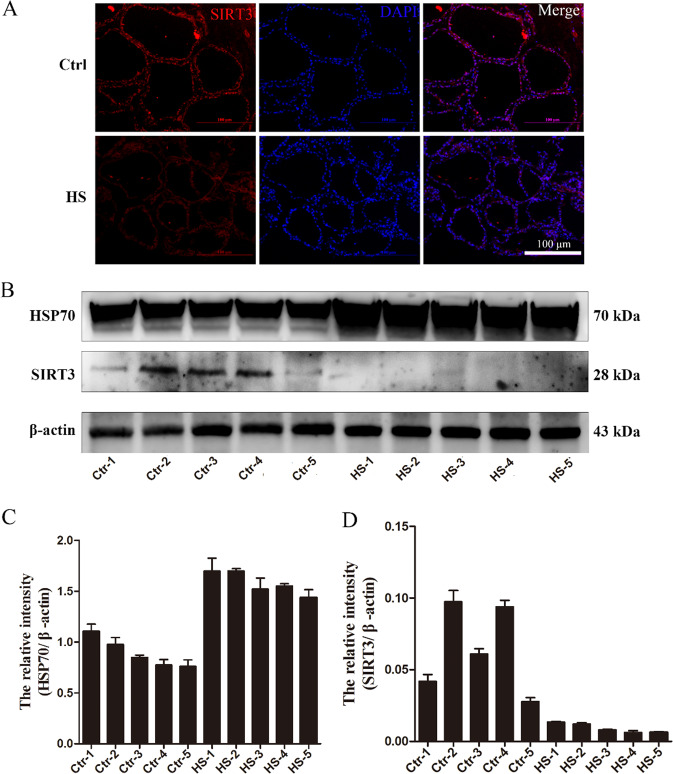
Expression of HSP70 and SIRT3 in normal and heat-stressed cow tissues. **A** Immunofluorescence staining to observe the changes in the expression of SIRT3 in dairy cow mammary tissue, scale bar: 100 μm. **B** Western blotting were performed to evaluate the protein expression of HSP70 and SIRT3 in the normal Holstein cattle and heat-stressed Holstein cattle. **C**, **D** ImageJ was used to quantify HSP70 and SIRT3 protein levels in the mammary tissues of normal Holstein cattle and heat-stressed Holstein cattle. Data are expressed as mean ± S.E.M.

First, SIRT3 expression was found in healthy bovine mammary tissue samples from the control group by immunofluorescence staining; however, SIRT3 expression was low in heat-stressed bovine mammary tissue samples (Fig. 1A). According to the results of western blotting, heat shock protein 70 (HSP70) was significantly upregulated, but SIRT3 was significantly downregulated in five heat-stressed bovine tissue samples compared with normal samples (Fig. 1B–D).

### SIRT3 regulates the cellular antioxidative defense capacity

**Fig. 2:**
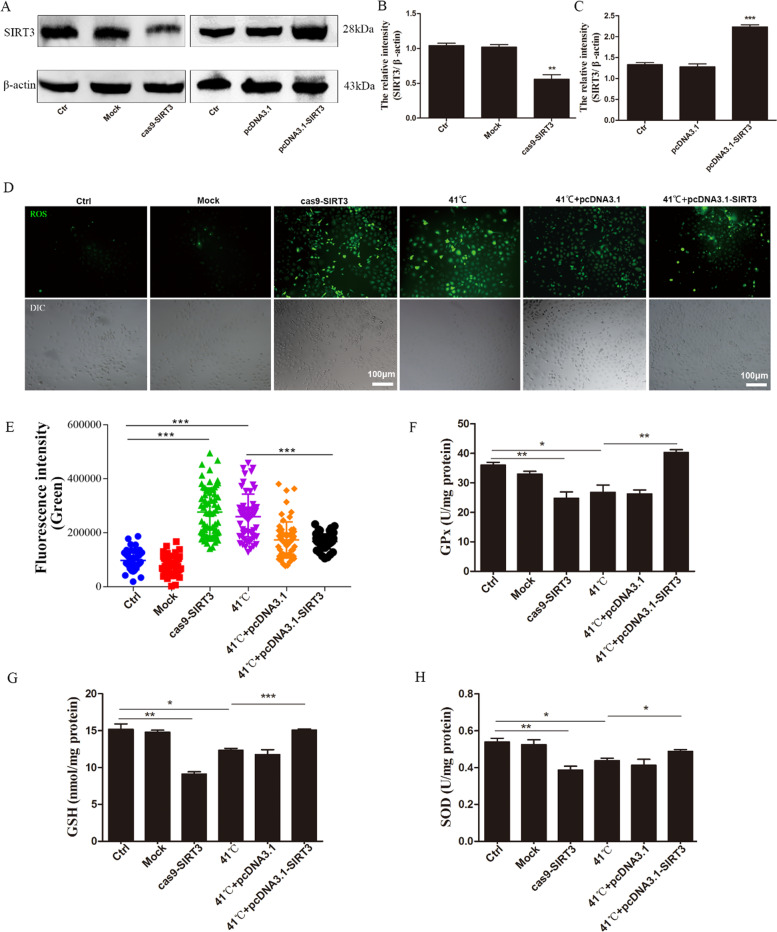
SIRT3 regulates ROS production and oxidase activity in BMECs under heat stress. **A**–**C** The knockdown and overexpression efficiency were evaluated using western blotting. **D** ROS is labeled with DCFH-DA. Scale bar: 100 µm, green: DCFH-DA. **E** The fluorescence intensity of green positive cells was quantified using Image J. **F**–**H** Kits were used to determine cellular antioxidant enzyme content. Ctr cells are not transfected; Mock (negative control) means to transfect cells with empty plasmid; 41 °C means that cells are cultured at 41 °C; 41 °C+pcDNA3.1 (control) means to use empty vector pcDNA3.1 transfection cells; 41 °C+pcDNA3.1-SIRT3 cells were transfected with pcDNA 3.1-SIRT3. Data are expressed as mean ± S.E.M, *P < 0.05, **P < 0.01, ***P < 0.001.

To verify the antioxidative function of SIRT3 in BMECs, clustered regularly interspaced short palindromic repeats/CRISPR-associated protein 9 (CRISPR/Cas9) technology was used to knock out SIRT3 and transfect it into a SIRT3 loss-of-function test. After Cas9-SIRT3 transfection, the level of SIRT3 protein was significantly downregulated (Fig. 2A, B). At the same time, in the overexpression experiment, compared with the control group, SIRT3 was significantly upregulated in cells transfected with pcDNA3.1-SIRT3 (Fig. 2A, C).

After heat stress treatment, ROS levels increased significantly compared with the control group, SIRT3 knockdown significantly increased the production of ROS, which had the same effect as heat stress, while the overexpression of SIRT3 significantly reduced the production of ROS induced by heat stress (Fig. 2D). We randomly selected five fields to quantify the number of ROS-positive cells after SIRT3 knockdown or overexpression in BMECs, and the results were consistent with our initial observations (Fig. 2E). Therefore, our data indicate that SIRT3 inhibits heat stress-induced ROS production in BMECs.

In addition, due to the increase and decrease in the production of ROS, the activity and concentration of antioxidant enzymes, such as superoxide dismutase (SOD), glutathione peroxidase (GPx), and glutathione (GSH), in the cell will change accordingly. SIRT3 knockdown significantly reduced the antioxidant enzyme activity, while SIRT3 overexpression reversed the change in the levels of cellular antioxidant factors caused by heat stress (Fig. 2F–H).

### SIRT3 regulates the level of mitochondrial membrane potential

**Fig. 3:**
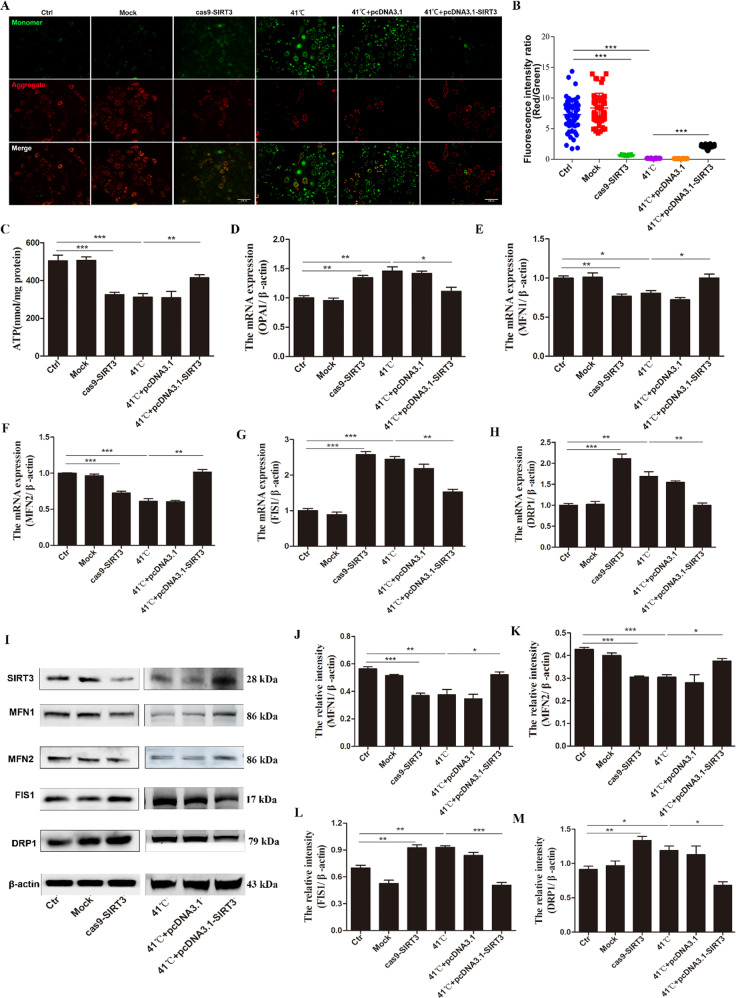
SIRT3 changes the level of mitochondrial membrane potential in heat-stressed BMECs and inhibits the mitochondrial damage induced by heat stress. **A** Observation of the mitochondrial potential using JC-1 staining. Red: JC-1 red fluorescence, green: JC-1 green fluorescence, scale bar: 100 μm. **B** The percentage of red/green fluorescence intensity was quantified using Image J. **C** Kits were used to determine the ATP content of cells. **D**–**H** RT-qPCR was used to detect the relative mRNA expression levels of OPA1 (**D**), MFN1 (**E**), MFN2 (**F**), FIS1 (**G**), and DRP1 (**H**). **I** The protein levels of MFN1, MFN2, FIS1, and DRP1 were determined by western blotting. **J**–**M** The quantification of the relative protein levels (performed using Image J) is shown under the band (protein/β-actin). Data are expressed as mean ± SEM (*n* = 3), *P < 0.05; **P < 0.01; ***P < 0.001.

The mitochondrial membrane potential was observed by JC-1 staining. After BMECs were treated with JC-1, normal mitochondria showed red fluorescence, and damaged mitochondria showed green fluorescence. As shown in Fig. 3A, B, SIRT3 knockdown resulted in an increase in the green fluorescence intensity and a decrease in the red fluorescence intensity, indicating that the mitochondrial membrane potential was reduced, whereas SIRT3 overexpression increased the mitochondrial membrane potential in heat-stressed cells. Due to the lack of mitochondrial membrane potential, mitochondria cannot produce sufficient ATP. We found that SIRT3 downregulated ATP content, whereas SIRT3 overexpression increased ATP content in heat-stressed cells (Fig. 3C).

### SIRT3 regulates mitochondrial dynamics

We also explored whether the loss of SIRT3 affects mitochondria, confirmed the relationship between SIRT3 and mitochondrial protection, and detected the mRNA and protein expression of genes related to mitochondrial division and fusion. First, the results of reverse transcription quantitative polymerase chain reaction (RT-qPCR) showed that the expression of optic atrophy protein 1 (OPA1) mRNA was significantly increased after SIRT3 knockdown (Fig. 3D). Mitochondrial division-related genes, such as *Fis1* and *Drp1*, showed the same trend (Fig. 3E, F), while SIRT3 overexpression significantly reduced the increase in expression caused by heat stress. The mRNA expression of mitochondrial fusion-related genes, such as *MFN1* and *MFN2*, was significantly reduced after SIRT3 knockdown. The overexpression of SIRT3, however, significantly increased the decreased expression of these genes due to heat stress (Fig. 3E, F). Subsequently, we performed western blotting and found that the levels of mitochondrial fusion-related proteins, including MFN1 and MFN2, were significantly reduced after SIRT3 knockdown but were significantly increased upon the overexpression of SIRT3. In contrast, the levels of mitochondrial division-related proteins, including Drp1 and Fis1, were significantly increased after SIRT3 knockdown, and SIRT3 overexpression significantly reduced this increase caused by heat stress, consistent with the RT-qPCR results (Fig. 3I–M).

### SIRT3 protects BMECs from oxidative damage

**Fig. 4:**
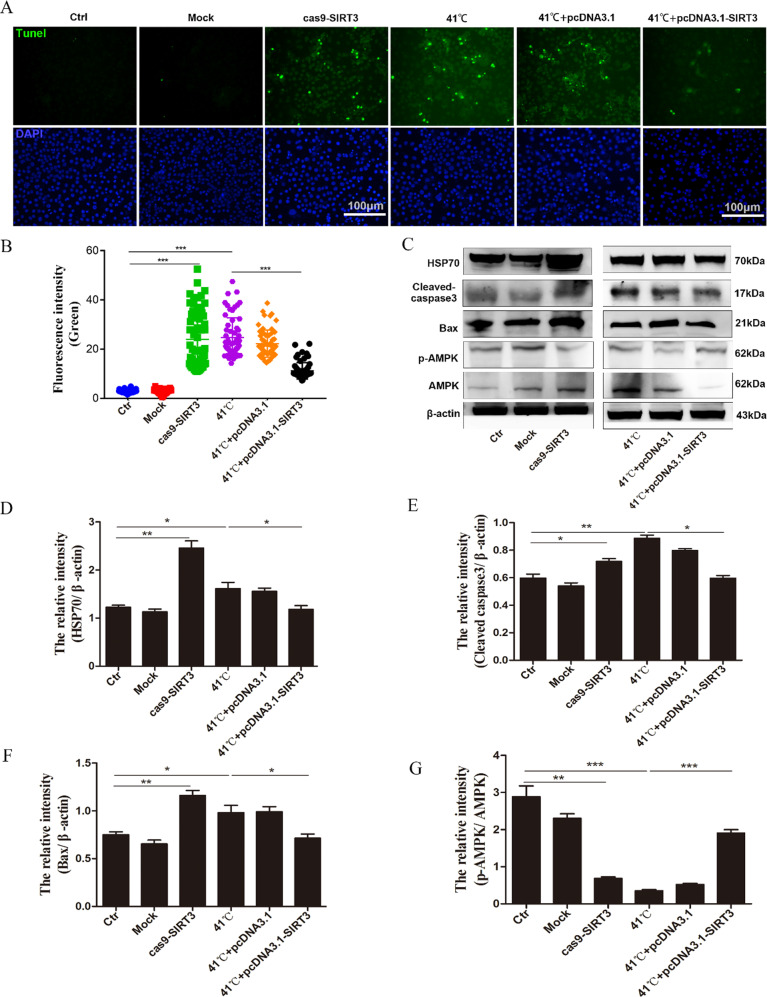
SIRT3 attenuates BMEC apoptosis induced by heat stress. **A** TUNEL staining was used to determine cell apoptosis. Green, TUNEL; blue: DNA; Scale bar: 100 µm. **B** The percentage of apoptotic cells was quantified by counting green positive cells. **C** The protein levels of HSP70, cleaved-caspase 3, Bax, p-AMPK, and AMPK were determined by western blotting. **D**–**G** The quantification of the relative protein levels is shown under the band (protein/β-actin). Data are expressed as mean ± SEM (*n* = 3), *P < 0.05; **P < 0.01; ***P < 0.001.

To observes the effect of SIRT3 on the apoptosis of BMECs, we performed terminal deoxynucleotidyl transferase-mediated dUTP-fluorescein nick end labeling (TUNEL) staining to detect the apoptosis of BMECs and found that, compared with the control group, SIRT3 knockdown significantly increased the number of apoptotic cells, while the overexpression of SIRT3 reduced cell apoptosis induced by heat stress (Fig. 4A, B). At the same time, western blotting results showed that SIRT3 knockdown can cause a significant upregulation of HSP70 as well as of the apoptosis markers, cleaved-caspase-3 and pro-apoptotic factor Bax. Overexpression of SIRT3, however, significantly reduced this upregulation of these proteins, induced by heat stress (Fig. 4C–F). This indicates that SIRT3 knockdown promotes oxidative stress and cell apoptosis.

To study the role of AMPK in the activation of SIRT3-mediated apoptosis, we first detected AMPK activation after knockdown and overexpression of SIRT3 by western blotting. SIRT3 knockdown is related to the inhibition of AMPK activation, which can be demonstrated by the decrease in AMPK phosphorylation. Overexpression of SIRT3 inhibited the upregulation of p-AMPK/AMPK caused by heat stress (Fig. 4C, H).

### SIRT3 regulates oxidative stress damage through AMPK activation

**Fig. 5:**
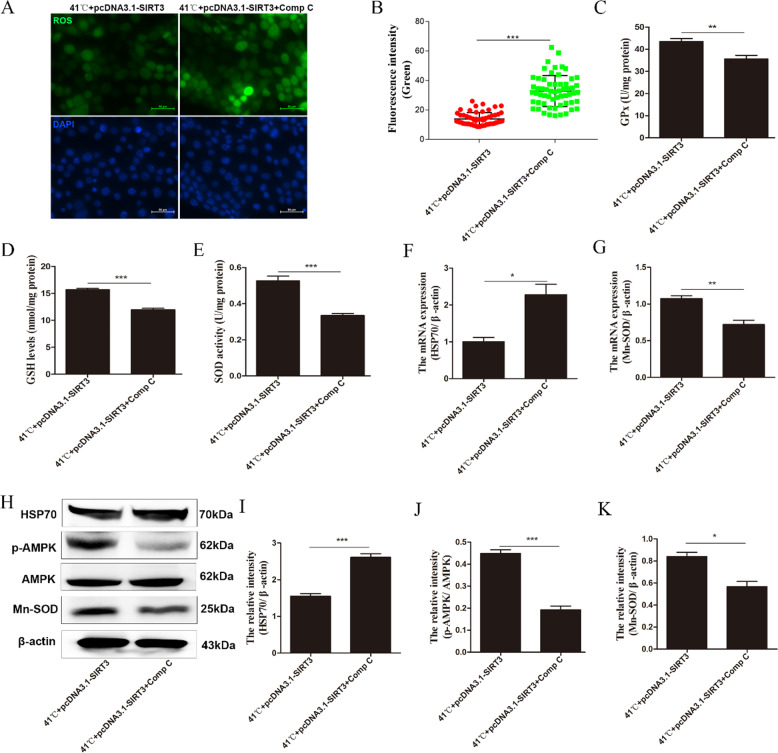
Inhibition of AMPK decreases the protective effect of SIRT3 on heat injury in BMECs. **A**, **B** After adding AMPK inhibitor, the level of intracellular ROS was detected. **C**–**E** After compound C treatment, the activities of antioxidant enzymes GPx (**C**), GSH (**D**), and SOD (**E**) were measured. **F**, **G** RT-qPCR was used to detect the relative mRNA expression levels of HSP70 and Mn-SOD. **H** Western blotting was used to detect the levels of Hsp70, p-AMPK, AMPK, and Mn-SOD after AMPK inhibition. **I**–**K** The quantification of the relative protein levels is shown under the band (protein/β-actin). Data are expressed as mean ± SEM (*n* = 3), *P < 0.05; **P < 0.01; ***P < 0.001.

To investigate whether the inhibition of AMPK triggers SIRT3 attenuation of oxidative stress, compound C was used to inhibit AMPK phosphorylation. After the addition of the inhibitor, the production of ROS increased significantly (Fig. 5A, B), and the activities of GPx, GSH, and SOD decreased significantly (Fig. 5C–E). Compound C not only inhibited the phosphorylation of AMPK (Fig. 5H, J) but also increased the inhibitory effect of SIRT3 on oxidative stress (Fig. 5F, H, I). The same pretreatment with compound C significantly inhibited the production of Mn-SOD after SIRT3 overexpression (Fig. 5H, K).

## Discussion

Holstein cattle in China are mostly produced in Northern Europe, and problems such as low fertility and milk quality decline are particularly prominent during summer, when heat stress in high [24]. The mammary tissue is the key site of milk synthesis and secretion. Under high temperature and during humid summers, an increase in oxidative stress caused by heat stress can seriously affect the function of the bovine breast tissue and significantly reduce the number and viability of BMECs [4, 25]. SIRT3 has various effects on mitochondrial energy metabolism [26] and plays a vital role in controlling oxidative stress in various cells and tissues. SIRT3 overexpression can improve mitochondrial performance in aged mice [27]. It is worth noting that some researchers have also found that, in ovarian cancer cells, activation of SIRT3 can reduce cellular energy stress [28]. A recent study showed that SIRT3 can protect against oxidative stress mediated by the expression of hepatitis B virus X protein [27]. Based on these antioxidant functions and benefits of SIRT3, we conducted this experiment to study the potential protective effect of SIRT3 against heat stress damage in BMECs. Our data lay the foundation for further in-depth studies on the molecular mechanism and regulation of oxidative stress in BMECs.

ROS are the initiators of oxidative stress in vivo, and mitochondria are the major ROS sources in vivo [29]. Excessive ROS accumulation leads to cell death by triggering the oxidization of polyunsaturated fatty acids in the cell membrane, a large number of unprotected protein sulfhydryl groups, and DNA bases [30]. Many studies have shown that appropriate levels of GSH can protect cells against the harmful effects of intracellular ROS [31]. As an endogenous antioxidant, GSH can reduce the levels of hydroperoxides and organic peroxides, including lipid peroxides, and protect cell membranes from free radical oxidative damage [32]. SOD is an important line of defense in the antioxidant defense system. SOD enzymes alternately catalyze the dismutation of superoxide (O^2−^) free radicals to ordinary molecular oxygen (O_2_) or H_2_O_2_, reducing ROS content to protect against oxidative stress [33]. Studies have shown that SIRT3 promotes ROS production by weakening the activity of SOD and reducing GSH content [34]. The current data show that the knockdown of SIRT3 increases net ROS production, while the overexpression of SIRT3 inhibits the net ROS production of BMECs after high-temperature treatment, as well as stabilizes oxidative stress factors (GPx, SOD, and GSH) in BMECs. Further, ROS can alter the mitochondrial membrane potential. Decreased mitochondrial potential impairs mitochondrial energy metabolism and decreases ATP synthesis [35, 36], thereby further aggravating the damage [37, 38]. In this study, we found that SIRT3 knockdown reduced the mitochondrial membrane potential and ATP production. These data confirm the role of oxidative stress in high-temperature-induced damage and clearly demonstrates the involvement of mitochondrial damage.

Mitochondria are dynamic organelles that continuously undergo division and fusion processes. Mitochondrial division is mainly regulated by mitochondrial dynamin-related protein 1 (DRP1) [39], mitochondrial fission protein (FIS1), and mitochondrial fission factor. Mitochondrial fusion is mainly regulated by mitochondrial fusion protein 1 (MFN1), MFN2, and OPA1 [40, 41]. Any disorder of mitochondrial dynamics changes the morphology of the mitochondria and affects its function [42–44]. Studies have shown that SIRT3 regulates renal ischemia–reperfusion injury (IRI) by enhancing mitochondrial fusion [45], SIRT3 protects liver cells from mitochondrial oxidative stress and effectively maintains mitochondrial integrity [46]. From previous studies, it can be concluded that SIRT3 is a key upstream regulator that maintains the balance of mitochondrial homeostasis. Our results showed that SIRT3 knockdown significantly increased the expression of FIS1 and DRP1 and reduced the expression of MFN1, MFN2, and OPA1, while SIRT3 overexpression showed the opposite trend under high-temperature-induced conditions. Through our experiments, we found that SIRT3 knockdown inhibited mitochondrial fusion and promoted mitochondrial division, while SIRT3 overexpression changed the trend induced by heat stress and affected mitochondrial function. This indicates that SIRT3 can reduce oxidative stress induced by high temperatures by mediating mitochondrial function.

In addition, the destruction of the integrity of the mitochondrial membrane through the activation of the caspase cascade eventually leads to cell apoptosis [47]. Oxidative stress promotes the expression of the apoptotic proteins’ caspase-3 and Bax, leading to apoptosis [48]. Bcl-2 family proteins exist in the outer mitochondrial membrane and participate in the regulation of mitochondrial function. The pro-apoptotic protein Bax translocates from the cytoplasm to the mitochondrial membrane and induces apoptosis [49]. The caspase family is the main component of apoptosis. Among them, effectors, such as caspase-3, are mainly activated by the proteolytic cleavage of promoters, following which they promote the degradation of intracellular targets, leading to cell death [50]. Studies have found that, in IRI mouse cardiomyocytes, melatonin can reduce mitochondrial oxidative stress and cardiomyocyte apoptosis by activating SIRT3, thereby improving cardiac IRI [51]. Our results show that SIRT3 knockdown upregulates HSP70, cleaved-caspase3, and Bax. At the same time, TUNEL staining also shows similar supporting results. Similar results have also been found in some previous studies on oxidative stress injury in animal models [52–54].

AMPK is responsible for monitoring the cell energy status [55] and is regulated by threonine 172 phosphorylation [56]. Activation of AMPK can reduce neuronal H/R damage and brain IR damage by inhibiting apoptosis and oxidative stress [57, 58]. In addition, the acetylated substrate of SIRT3 directly phosphorylates and activates AMPK [59, 60]. Therefore, here we investigated whether SIRT3 could reduce the high-temperature-induced oxidative stress damage in BMECs through the AMPK pathway. The results showed that the p-AMPK/AMPK ratio was significantly reduced after SIRT3 knockdown and that the overexpression of SIRT3 could change the high-temperature-induced ratio reduction. Following the inhibition of AMPK, the p-AMPK/AMPK expression of BMECs was significantly reduced; the activities of SOD, GSH, and GPx were significantly downregulated; the content of ROS was significantly increased; the expression of HSP70 was significantly increased; and the expression of Mn-SOD was downregulated. These results indicate that SIRT3 may reduce oxidative stress damage by increasing AMPK phosphorylation.

**Fig. 6:**
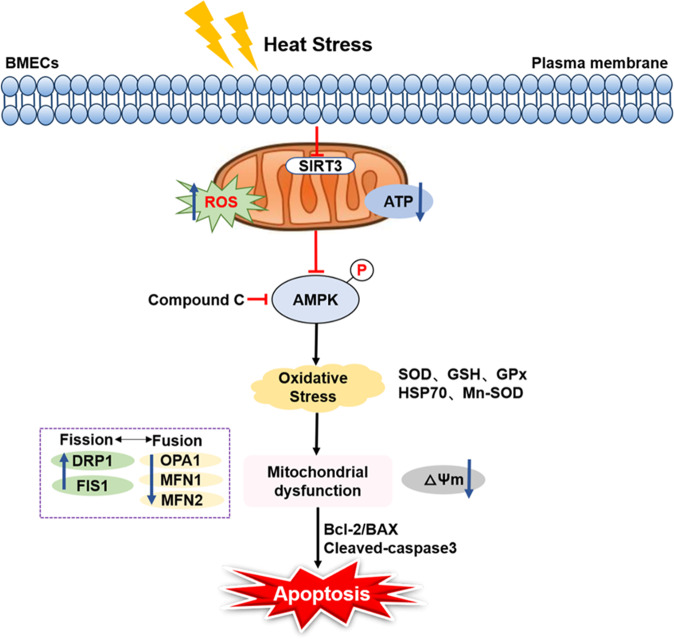
The potential functional mechanism of SIRT3 in heat stress-induced BMECs. Heat stress induces low expression of SIRT3, inhibits AMPK phosphorylation, activates cell oxidative stress and mitochondrial damage, and ultimately leads to cell apoptosis.

In summary, high ambient temperatures cause low expression of SIRT3, inhibit AMPK phosphorylation, and then induce oxidative stress and mitochondrial damage, ultimately leading to cell apoptosis. However, SIRT3 overexpression can reverse the damage caused by heat stress in BMECs (Fig. 6). Therefore, SIRT3 plays an important role in regulating the anti-heat stress defense system.

## Materials and methods

### Collection of cow mammary tissue sample

The protocol of the present study was approved by the Institutional Animal Care and Use Committee of Nanjing Agricultural University. We collected five normal and five heat stress mammary tissue samples from Chinese Holstein cows in a local slaughterhouse for protein extraction and immunofluorescence staining. Within 20 min after slaughter, a sample of breast tissue was quickly collected and divided into two parts, one of which was stored in liquid nitrogen until analyzed, and the other was fixed in 4% paraformaldehyde for use.

### Cell culture treatment and heat stress model

The BMEC line was supplemented with 10% fetal bovine serum (Bioindustry, catalog: 04-001-1ACS) in Dulbecco’s Modified Eagle’s Medium/F-12 Medium (Basal Media, catalog number: A210808, Shanghai) and bovine serum (Bioindustry, catalog: 04-001-1ACS, Shanghai) culture. All the cells were inoculated into T25 cell culture flask and incubated with 5 mL medium for 24 h. The incubator contained 95% air and 5% CO_2_. The cells needed for the experiment were inoculated into the culture plate.

Then the cells were incubated at 37 °C (Control) and 41 °C (HS) to establish a heat stress model. The control group was cultured in an incubator at 37 °C. Cells were cultured at 41 °C in order to represent a severe heat stress, since environmental conditions can reach 41 °C, and in summer, extreme conditions may exceed this temperature. Finally, cells were cultured at 41 °C for 3 h to establish a heat stress model.

### Transfection

CRISPR/Cas9 gene-editing technology was used to target SIRT3 gene knockdown, and pcDNA3.1 was used as the vector for recombinant cyclization to construct pcDNA3.1-SIRT3 overexpression vector. BMECs were seeded in a 6-well plate and cultured for 24 h until they reached 50–60% confluence. BMECs was transfected with plasmids using Lipofectamine 3000 (Invitrogen, Carlsbad, Cat: L3000008). Cells were transfected with 1.7 μg knockdown plasmid Cas9-SIRT3 and empty vector. Cells were transfected with 2 μg of pcDNA3.1-SIRT3 for SIRT3 overexpression or an empty vector (control plasmid pcDNA3.1). Transfection efficiency was determined via RT-qPCR and western blotting. Table S1 lists the SIRT3 single-guide RNA sequence synthesized by Nanjing Gen Script Biotech in Supplementary Materials.

### Mitochondrial membrane potential measurement

The mitochondrial membrane potential was measured with JC-1. First, cultured breast epithelial cells were spread on a 24-well plate for transfection and cultured at 37 °C and 5% CO_2_ saturated humidity for 48 h. In all, 1 mL JC-1 staining working solution was added to treat the cells and incubated them in the dark in a 37 °C incubator for 20 min, then washed twice with cold JC-1 staining buffer (1×), 2 mL cell culture medium was added, and the mitochondrial membrane potential was observed under a fluorescence microscope.

### GSH, GPx, and SOD detection

After cell processing, a kit was used to detect GSH (Beyotime, catalog: S0056, Shanghai), GPx (Beyotime, catalog: S0051, Shanghai), and SOD (Beyotime, catalog: S0103, Shanghai), and the assay was performed according to the kit manufacturer’s instructions.

### RNA extraction and RT-qPCR

The instructions of the RNA Extraction Kit (BioTeke) was followed to isolate total RNA from the cells and extract the appropriate RNA for reverse transcription. The reaction was divided into two steps. The first step is to remove genomic DNA (RNase-free ddH_2_O to 16 μL, 4 × gDNA wiper Mix 4 μL, total RNA 1000 ng) reaction program, 41 °C for 2 min, and the second step reverse transcription reaction system (5 × HiScript II qRT SuperMix 4 μL, the first step is the reaction solution (16 μL) reaction program, 50 °C 15 min, 85 °C 5 min, get cDNA, and quantitative mRNA expression by real-time PCR.

Using the method of comparing Ct (2^−ΔΔCt^) values can normalize the expression levels of all genes with the endogenous reference gene β-Actin. The primer sequences are listed in Table S2 of Supplementary Materials.

### Western blotting

Total protein was extracted using a kit from Sangon Biotech (catalog number: FA25DA0004, Shanghai) according to the manufacturer’s instructions. Total protein with lysis buffer containing protease inhibitors was extracted, incubated on ice for 30 min, shaken once every 5 min, collected in a 1.5 mL centrifuge tube after completion, and centrifuged at 4 °C (12,000 × *g*, 10 min). The enhanced BCA Protein Assay Kit (CWBIO, catalog number: CW0014S, Jiangsu) was used to determine the protein concentration of the sample. Protein denaturation is performed according to the protein concentration. The same volume of denatured protein was separated with sodium dodecyl sulfate-polyacrylamide gel electrophoresis gel, and then transferred to polyvinylidene difluoride membrane. After blocking with 5% skimmed milk for 1 h at room temperature, target band was cut and incubated with the next antibody overnight at 4 °C (SIRT3, 1:1500 (Proteintech, catalog: 10099-1-AP, Wuhan), β-actin, 1:2000 (Affinity, catalog: AF7018, Shanghai), MFN1, 1:1000 (Proteintech, catalog: 13798- 1-AP, Wuhan), MFN2, 1:2000 (Proteintech, catalog: 12186-1-AP, Wuhan), FIS1, 1:2000 (Proteintech, catalog: 10956-1-AP, Wuhan), Drp1, 1:2000 (Proteintech, catalog: 12957-1-AP, Wuhan), HSP70, 1:2000 (Proteintech, catalog: 10995-1-AP, Wuhan), cleaved-caspase-3, 1:500 (Affinity, catalog: AF7022, Shanghai)), BAX, 1:2000 (Proteintech, catalog: 50599-2-lg, Wuhan), p-AMPK, 1:1000 (Cell Signaling, catalog:#2535, Shanghai), AMPK, 1:1000 (Cell Signaling, catalog: #5832, Shanghai), Mn-SOD, 1:2000 (Proteintech, catalog: 24127-1-AP, Wuhan)). After washing three times with TBST, the strips were incubated with a secondary antibody containing horseradish peroxidase for 2 h. Then it was washed three times with TBST and developed using ECL Chemiluminescent Substrate Kit (Affinity, catalog number: 1824c03, Shanghai). ImageJ software was used to analyze all bands by densitometry and normalize the relative protein intensity values.

### ROS assay

The treated cells were loaded with probe 2′,7′-dichlorodihydrofluorescein diacetate (DCFH-DA) (Beyotime, cat: S0033S, Shanghai) and washed the cells twice with phosphate-buffered saline (PBS), and probes were diluted 1:1000 in serum-free medium, incubated in 37 °C cell incubators in the dark for 20 min, and mixed upside down every 3–5 min uniformly, and then washed three times with serum-free medium to remove the DCFH-DA that had not entered the cells and then the cells on glass slides were examined with a fluorescence microscope (Olympus, Tokyo, Japan), and the fluorescence intensity of 60 randomly selected cells in three experiments were analyzed using Image J. ROS oxidize non-fluorescent DCFH into fluorescent DCF, so the amount of fluorescent DCF indicates the level of ROS in the cells. The percentage of ROS is determined based on the counts in five randomly selected fields of view.

### Immunofluorescence staining

The breast tissues fixed with paraformaldehyde were dehydrated, and frozen sections (4 μm) were made. After processing, the buffered glycerol mounts are protected from light. Under the same magnification and parameters, five visual fields were randomly selected for each frozen section of breast tissue to be photographed.

### Apoptosis detection

The cell apoptosis detection is carried out in accordance with the instructions of Beyotime’s one-step TUNEL cell apoptosis detection kit (C1086). The detailed steps are as follows: After 48 h of transfection, the adherent cells were washed once with PBS, the cells were fixed with 4% paraformaldehyde for 30 min and washed once with PBS, and PBS containing 0.3% Triton X-100 was added and incubated for 5 min at room temperature. During the incubation period, TUNEL detection solution was prepared. At the end of incubation, the cells were first washed with PBS twice and 50 μL TUNEL detection solutions was added to each treatment hole, and incubated at 37 °C for 60 min in the dark. Later, the cells were washed three times with PBS and the mount with anti-fluorescence quenching mounting solution was mounted and observed under a fluorescence microscope.

### Statistics and analysis

The average value of the measured data is expressed as ±SEM. All experiments were repeated three times for each group. Using the software GraphPad Prism 7.0.1 (La Jolla, California, USA), all results were analyzed by one-way analysis of variance to determine the significant differences between the groups. *P* < 0.05 was examined statistically significant.

## Supplementary information

Table S1

Table S2

## Data Availability

The datasets for the current study are available from the corresponding author upon reasonable request.
