# Supplementary material for: SIRT3 protects bovine mammary epithelial cells from heat stress damage by activating the AMPK signaling pathway
Source: Cell Death Discov. 2021 Oct 21;7:304. doi: 10.1038/s41420-021-00695-7 (PMC8531291; doi:10.1038/s41420-021-00695-7)
Supplement: Supplementary file 1 — Table S1 [file 41420_2021_695_MOESM1_ESM.docx]

Table S1. The primer sequences of sgRNA.

| sgRNA | Primer Sequences (5’-3’) |
| --- | --- |
| sgRNA1 | F: CCCAGTCCAACCTTTGTCGT  R: aaacGCGAGGTGCGCGGGCGTGGAC |
| sgRNA2 | F: CACCGCTGAAGTCTGGGATGCCGCT  R: aaacAGCGGCATCCCAGACTTCAGC |
| sgRNA3 | F: CACCGCTCCGATTGCTACACGAGAA  R: aaacTTCTCGTGTAGCAATCGGAGC |
