# Supplementary material for: SIRT3 protects bovine mammary epithelial cells from heat stress damage by activating the AMPK signaling pathway
Source: Cell Death Discov. 2021 Oct 21;7:304. doi: 10.1038/s41420-021-00695-7 (PMC8531291; doi:10.1038/s41420-021-00695-7)
Supplement: Supplementary file 2 — Table S2 [file 41420_2021_695_MOESM2_ESM.docx]

Table S2. Primer sequences of mRNA

| Genes | Forward | Reverse |
| --- | --- | --- |
| SIRT3  MFN1  MFN2  OPA1  DRP1  FIS1  Mn-SOD  β-Actin | 5’-TCCCTGACTCAAAGCTCGTT-3’  5’- ACAGCACATGGAAAGATGCC-3’  5’-AGAGGGCTCAGAGGAGAAGA-3’  5’-TGGTGCTTGTTGACCTACCA-3’  5’-CAGAGAGCTCATCCTTCGGT-3’  5’-CACAGAACAACCAGGCCAAA-3’  5’-AATCACAGCATCTTCTGGACAA-3’  5’-TCACCAACTGGGACGACA-3’ | 5’-CCCGAAGAACACGATGTCAG-3’  5’-CCCTGTGCTTTGTGCTTTCT-3’  5’-CTTGAGAAGCGGACACTTGG-3’  5’-CGTTCAGCATCCACAGATC-3’  5’-TGCGACCATCTGGATCTACC-3’  5’-TGCCGTCTCCTTCAGGATTT-3’  5’-ACCAACAGATACAGCAGTCAAC-3’  5’-GCATACAGGGACAGCACA-3’ |
